# A Review on the Thermal Characterisation of Natural and Hybrid Fiber Composites

**DOI:** 10.3390/polym13244425

**Published:** 2021-12-16

**Authors:** Jorge S. S. Neto, Henrique F. M. de Queiroz, Ricardo A. A. Aguiar, Mariana D. Banea

**Affiliations:** Federal Centre of Technological Education in Rio de Janeiro (CEFET/RJ), Rio de Janeiro 20271-110, Brazil; jorge.neto@aluno.cefet-rj.br (J.S.S.N.); henrique.queiroz@aluno.cefet-rj.br (H.F.M.d.Q.); ricardo.aguiar@cefet-rj.br (R.A.A.A.)

**Keywords:** natural fiber reinforced composite material, thermal analysis, thermogravimetric analysis (TGA), differential scanning calorimetry (DSC), differential mechanical thermal analysis (DMA)

## Abstract

The thermal stability of natural fiber composites is a relevant aspect to be considered since the processing temperature plays a critical role in the manufacturing process of composites. At higher temperatures, the natural fiber components (cellulose, hemicellulose, and lignin) start to degrade and their major properties (mechanical and thermal) change. Different methods are used in the literature to determine the thermal properties of natural fiber composites as well as to help to understand and determine their suitability for a certain applications (e.g., Thermogravimetric analysis (TGA), differential scanning calorimetry (DSC), and differential mechanical thermal analysis (DMA)). Weight loss percentage, the degradation temperature, glass transition temperature (*T_g_*), and viscoelastic properties (storage modulus, loss modulus, and the damping factor) are the most common thermal properties determined by these methods. This paper provides an overview of the recent advances made regarding the thermal properties of natural and hybrid fiber composites in thermoset and thermoplastic polymeric matrices. First, the main factors that affect the thermal properties of natural and hybrid fiber composites (fiber and matrix type, the presence of fillers, fiber content and orientation, the treatment of the fibers, and manufacturing process) are briefly presented. Further, the methods used to determine the thermal properties of natural and hybrid composites are discussed. It is concluded that thermal analysis can provide useful information for the development of new materials and the optimization of the selection process of these materials for new applications. It is crucial to ensure that the natural fibers used in the composites can withstand the heat required during the fabrication process and retain their characteristics in service.

## 1. Introduction

The application of composites has continuously increased across many industries, in particular in the automotive and aerospace industries where lower weight and high resistance are key factors. The most commonly used fibers that attend these requirements are carbon and glass fibers [1,2,3]. However, nowadays, the industry is seeking new desirable characteristics of composite materials, such as renewability, eco-friendliness, and low cost. Consequently, there has been great interest in research and innovation in natural fiber composites owing to the advantages of these materials compared to their synthetic fiber counterparts (i.e., lower environmental impact and lower cost), supporting their potential across a wide range of applications in several industrial sectors [4,5,6,7,8,9]. The natural fiber-reinforced composites (NFRCs) are used mainly in non-structural car body parts, such as door panels, package trays, hat racks, instrument panels, internal engine covers, sun visors, boot liners, oil air filters, and even progressing to more structurally demanding parts, such as seat backs and exterior underfloor paneling [10,11]. Nowadays, most of the automotive makers, such as Audi, Volkswagen, Toyota, Daimler-Benz, Volvo, Ford, etc., use NFRCs to produce components. The continually growing demands for lightweight and fuel-efficient vehicles will further push the growth of NFRCs in the automotive market. There are other exciting market trends going forward in many different industries. For example, tri-dimensional hybrid natural fiber reinforcement preforms have been used recently by sports car manufacturers, such as Porsche and even McLaren in Formula 1 [12]. Other applications of NFRCs include sport equipment, musical instruments, aerospace, construction industry [12,13,14], and ballistic armour [15,16].

Several types of natural fibers are currently used in industry, such as jute, sisal, oil palm, kenaf, and flax, which are well established in the global market with a well-defined production line. However, new promising natural fibers are being discovered and used on a smaller scale or are still being used only for research. This is the case of the buriti and curauá fibers, for example, that still need some improvements in their production line to be more commercially affordable and reach widespread use [17,18]. They are used as reinforcement fibers in thermoset or thermoplastic polymeric matrix in a variety of applications [19]. Depending upon the matrix type, NFRCs are categorized into completely biodegradable or partially biodegradable composites.

The growing importance of natural fiber reinforced composites is reflected by the increasing number of publications (e.g., reviews, patents, book chapters, and books) during the recent years [4,5,20,21,22,23,24,25]. Therefore, it is important to study their thermal and mechanical behaviour in order to utilize their full potential. The thermal stability of natural fiber composites is a relevant aspect to be considered as the processing temperature plays a crucial role in the fabrication process of the composites. At higher temperatures, the natural fiber components (i.e., cellulose, hemicellulose, and lignin), start to degrade and the major properties (mechanical and thermal) of the composite change. Intense research efforts are continuously made and some of the shortcomings of NFRCs were addressed by recent advancements in fiber treatment and modification, exploration of new natural fibers, and hybridization. The fiber modification techniques provide improved fiber–matrix interfacial adhesion, improved fiber roughness, and wettability and depend on the particular fiber/matrix used and the composite application, while the hybridization methods provide flexibility in fiber selection for the material properties according to the end-use application requirements.

Even though there are many recent review articles concerning the use of natural fibers in the production of natural hybrid composites [10,23,26,27,28,29,30,31,32,33,34], one topic that was not covered in any significant detail relates to the thermal characterisation of NFRCs. This paper provides an overview of the recent advances in the thermal properties of natural and hybrid natural fiber composites in thermoset and thermoplastic polymeric matrices. First, the main factors that affect the thermal properties of natural and hybrid fiber composite materials (fiber and matrix type, the presence of additive fillers, fiber content and orientation, the treatment of the fibers, manufacturing process, and type of loading) are briefly presented. Further, the methods used to determine the thermal properties of natural and hybrid composites are discussed. Finally, some conclusions and critical challenges and future perspectives and research activities are summarized.

## 2. Factors That Affect the Thermal Properties of Natural and Hybrid Composites

The main factors that affect the thermal properties of natural and hybrid fiber compo-site materials are: fiber and matrix type, the presence of additive fillers, fiber content and orientation, the treatment of the fibers, manufacturing process, and type of loading. In this section, these factors are briefly discussed.

### 2.1. Type of Fiber

**Figure 1:**
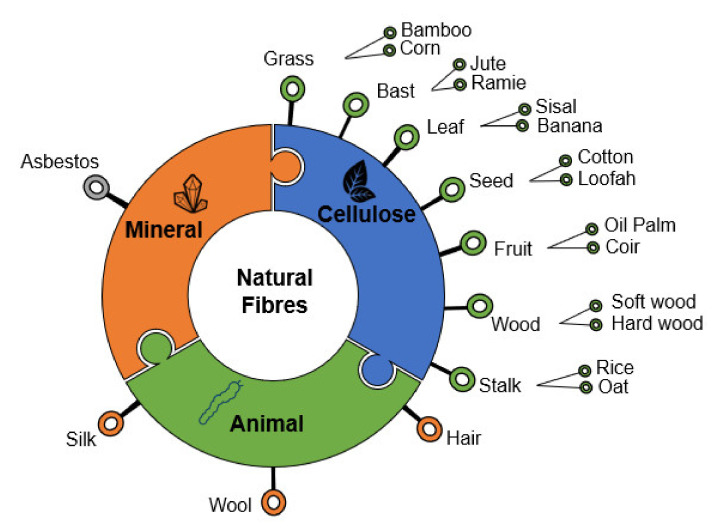
Schematic of natural fibers classification.

The natural fibers are promising renewable alternatives to the traditional human-made fibers (i.e., glass and carbon fibers) in composite materials. Their consumption is increasing in different industrial sectors, such as aeronautical, automotive, civil, and naval, with an average annual growth of 7.5% and an expected global market demand around 35 million tons by 2022 [10]. The natural fibers can be divided into three main groups regarding their origin: mineral, animal, and cellulose/lignocellulose (see Figure 1). Mineral-based fibers were widely used in composite materials. However, their use presented many human health issues (carcinogenic parts that can be inhaled or ingested), nowadays being forbidden in many countries worldwide. The animal fibers present lower mechanical properties compared to the cellulose fibers, except for the silk fiber that presents high tensile strength [10]. However, the silk fibers are quite expensive and are more used in the textile industries [10]. The cellulose/lignocellulose fibers are the most used natural fibers due to their relatively low price and higher mechanical properties compared to other natural fibers and are the focus in the present review. They can also be subdivided into different categories corresponding to the plant part that originated them, as can be seen in Figure 1. Depending on the length, orientation, and type of natural fiber, randomly oriented (short), unidirectional, and woven fabrics are usually used as reinforcements in thermoset and thermoplastic matrices [35].

The thermal stability of any natural fiber is the maximum temperature at which a fiber decomposes [36]. It is known that various constituents of the natural fiber (i.e., hemicellulose, cellulose and lignin) decompose at different temperatures which leads to the complete decomposition of the fiber as a whole [37]. It was reported that, lignin starts degrading at a temperature around 200 °C, while hemicelluloses and cellulosic constituents degraded at higher temperatures [38]. Usually, the thermal degradation of lignin and cellulose occurs in the temperature range of 300–450 °C [39]. The thermal stability of the natural fiber can be enhanced by removing a certain proportion of hemicelluloses and lignin constituents. However, fibers with lower lignin content are susceptible to thermal damage and often tend to damage secondary cell walls [40]. Other volatile or partially stable constituents (i.e., pectin, waxes, and water-soluble substances), may also be found in cellulose fibers. Thus, the thermal properties of the natural fiber leads to critical issues for the processing of natural fiber-reinforced thermoplastic composite products. For instance, the automotive interior part made of natural fiber reinforced composites can be affected by the high temperature inside the vehicle during the hot season. In addition, the thermal stability of these composites is a critical issue because of the poor heat resistance during a fire. If the natural fibers are exposed to temperatures higher than 250 °C, they have a very high probability of burning, thus compromising the structural integrity of the component [41].

To summarize, the thermal stability of natural fiber composites is a relevant aspect to be considered as the processing temperature plays a crucial role in the fabrication process of the composites. Intense research efforts are continuously made and some of the shortcomings of NFRCs were addressed by recent advancements in fiber treatment and modification, by the exploration of new natural fibers and hybridization.

### 2.2. Surface Treatment of Natural Fibers

The surface treatment of natural fibers is usually performed to enhance their mechanical and thermal properties, before using them to manufacture a composite material. Fiber surface modification can improve the fiber–matrix interfacial bonding, roughness, and wettability and decrease the moisture absorption of the fibers [5,42,43]. As a result, better mechanical and thermal properties of the resulting composites can be achieved. The main surface modification methods used for natural fibers are: physical and chemical treatments.

The main physical treatments used to modify the surface of natural fibers are as follows: plasma, corona, ultraviolet (UV), heat treatments, electron radiation, and fiber beating. These treatments improve the adhesion between the fiber and matrix by changing the surface properties of the fibers without changing their structural composition [44,45].

The chemical treatments are a common way of increasing the interface adhesion between the fiber–matrix by chemical bonding or mechanical interlocking at the interface and decreases the water absorption of the fibers. This is achieved by using compounds that promote adhesion by chemically coupling the fibers to the matrix, such as: sodium hydroxide [46], silane [47], acetic acid [48], Benzoyl Chloride [49], maleated coupling agents [44], isocyanates [5], peroxide [50], and stearic acid [51].

Numerous researchers have investigated the effect of the fiber treatments on the thermal properties of NFRCs [36,52,53,54,55,56,57,58,59,60,61,62,63,64,65,66,67,68,69,70,71,72,73,74]. In general, fiber pre-treatments increased the thermal stability of the resulting NFRCs, mainly due to the removal of the waxy layers and other impurities from the fiber surface, as well as increasing the crystallinity [75,76,77]. However, some researchers found that surface treatments of natural fibers led to a decrease in degradation temperatures, signifying weakened thermal stability of the composites [78,79]. In addition, in some cases, the gain in thermal stability negatively affected the mechanical properties of composites. It was stated that the balance in improvements in thermal properties depends on fiber treatment type and time. It was also shown that the combination of treatments (mixed treatments) promoted increased thermal stability [80]. Table 1 summarizes the results from several recent studies on the effect of fiber treatments on the thermal properties of natural fiber composites.

To summarize, using natural fibers with low lignin content leads to better thermal performance of composites. The thermal stability of fibers can be enhanced by removing a certain proportion of hemicelluloses and lignin constituents by different chemical treatments. The fiber modification techniques provide improved fiber–matrix interfacial adhesion, improved fiber roughness, and wettability and depend on the particular fiber/matrix used and the composite application. The thermal degradation of natural fibers is an important issue in the development of natural fiber reinforced composites in both manufacturing and for the use of these materials in service. In general, it was stated that in order to avoid the thermal degradation of developed NFRCs, the processing temperature must be kept below 250 °C.

### 2.3. Type of Matrix

In natural fiber reinforced polymer composites, both thermoplastic and thermoset polymers are used as matrices. There are several types of thermoplastics such as polypropylene (PP), polyethylene (PE), nylon, polystyrene (PS), polyvinyl chloride (PVC), poly (methyl methacrylate) (PMMA), polytetrafluoroethylene (PTFE), polylactic acid (PLA), and acrylonitrile butadiene styrene (ABS), which can be used as matrix with natural fiber composites. When heated, thermoplastics soften and can be remoulded without significant degradation into a variety of products. Thus, their most important characteristic is the recyclability. They present other benefits, such as being lightweight, durable materials and they are reasonably priced [41,95]. The processing of natural fiber reinforced thermoplastic composites usually involves extrusion of materials at melt temperatures followed by shaping operations (i.e., injection molding and thermoforming). However, due to some degree of incompatibility of nonpolar hydrophobic PP and PE with polar and hydrophilic natural cellulose fibers, these types of resins are typically not used as matrices for NFRCs.

PLA is one of the most used thermoplastic polymers as a matrix in natural fiber composites. PLA is a widely used commercial biodegradable polymer, which belongs to one of the thermoplastic aliphatic polyesters produced from natural resources like corn, rice, and sugar beets [96]. PLA presents good stiffness, high strength, and low elongation at break, which make it an ecologically friendly material for composite applications [97]. However, the mechanical and thermal properties of PLA are strongly related to crystallinity. The wide application of PLA is limited by its low crystallization degree/rate, high brittleness and low thermal resistance (its thermal resistance is limited by its low glass transition (60 °C) [98]). The thermal properties of natural fiber reinforced thermoplastic composites were studied by numerous researchers [41,99,100,101,102,103,104,105,106].

On the other hand, the thermoset matrices (epoxy, urethane, vinyl ester, phenolic, polyester, polyimide, polyurethane (PU)) can hardly be recycled or change the shape once the polymerization (curing) is concluded. The main thermoset resins used for natural fiber composites are epoxy and unsaturated polyesters [107]. Finally, biodegradable polymer matrices (elastomers, thermosets, and thermoplastics) are also used in the manufacture of NFRCs and combining these matrices with natural fibers produces “green composites” or “bio-composites”. Biodegradable thermosets have gathered more interest in producing green composites in comparison to thermoplastics [108].

There is an intense effort in the scientific community to increase the performance of the thermoplastic biopolymers (mainly based on PLA matrices). Some examples of studies about the thermal properties of natural fiber reinforced green composites will be discussed in the next sections [109]. Some researchers performed comparative studies on the mechanical properties of epoxy and polyester composites reinforced with hemp fiber or sisal, jute, and curauá [19], in order to discover which of these thermoset matrices is more adequate for novel NFRCs to be used in technological applications. In general, the epoxy based NFRCs revealed superior mechanical and thermal properties to those of polyester NFRCs. In another study, the thermal properties of different NFRCs based on thermoplastic matrices (Acrylonitrile butadiene styrene (ABS), high impact polystyrene (HIPS), and high density polyethylene (HDPE)) reinforced with banana fiber were compared [110]. However, all these matrices have different chemical structures and undergo different reactions with the surface of natural fibers in composites. The thermal stability of composites is directly related to the crystallinity of the matrix and fiber reinforcements (high crystallinity improve the heat resistance).

### 2.4. Incorporation of Fillers, Fiber Content and Orientation

It was shown in the literature that the presence of fillers, fiber content and orientation, and the manufacturing process of the composites also affects the thermal properties of composite materials [105,111,112,113,114,115,116,117,118,119,120,121,122,123,124,125,126,127,128,129,130,131,132].

The nature of fillers can be organic, inorganic metallic, and ceramic and they can be in macro, micro, or nano scale [133]. In general, superior thermal properties can be achieved with fillers at nano-scale, at low reinforcement concentration (usually <10 wt%). For instance, Ramesh et al. [114] investigated the effect of MMT clay filler in the thermal properties of Kenaf fiber-reinforced PLA composite. The kenaf fibers were treated with 6% of NaOH solution for 3 h, and the amount of MMT used was 1%, 2%, and 3%. It was found that the presence of MMT clay improved the thermal stability. The MMT clay acts as a barrier, preventing the PLA polymer matrix from volatilizing. The segmental mobility of the polymer networks between the clay layers is limited, resulting in better thermal stability properties. Arulmurugan et al. [128] studied the effect of barium sulfate (BaSO_4_) on the thermal properties of an aloevera/flax hybrid composite. The fibers were subjected to chemical pre-treatment (sodium hydroxide and potassium permanganate) with 10% and 5% for 1 h, respectively. Then, they were treated with sodium laurel sulphate with 2% for 30 min. The authors report that the increase in BaSO_4_ in the pure and hybrid composite increased the thermal resistance of hybrid natural reinforced polymeric. Moreover, the maximum peak for the treated composites shows an increment with the addition of BaSO_4_ in its structure. In a different study, Bhoopathi et al. [134], investigated the influence of eggshell nanoparticles on the thermal properties of hemp reinforced composites. The natural fibers were treated by mercerization (5% of NaOH for 5 h at room temperature). The studied cases were: Hemp without eggshell (0%ESP), hemp + 7% eggshell (7%ESP), hemp + 14% eggshell (14%ESP), hemp + 21% eggshell (21%ESP). The authors report that increasing the nanoparticle percentage increased the degradation temperature of the composites when compared to the unmodified composite samples. The best case found was the 14%ESP, where the maximum exothermic peak value of degradation temperature onset was 411.6 °C while for the unfilled case it was 326.2 °C. Gouda et al. [135] studied hybrid composites with bamboo and GNP filler and found that the hybrid composites exhibited improved thermal conductivity. DMA results showed that the *T_g_* of the hybrid composite materials lay in the range of 90–95 °C.

Sumesh et al. [116] investigated the thermal properties of cellulose micro filler (CMF) filled pineapple (PA)/flax (FL) hybrid composites and showed that the endothermic enthalpy and the endothermic peak increased due to the addition of CMF to the PA/FL composite. The improvement in the endothermic peak was from 92.99° to 114.94° for the 2% CMF and 30% PA/FL specimens, while the endothermic energy increased from 492.34 Jg^−1^ to 499.39 Jg^−1^. The 3% CMF with 35% PA/FL specimens presented an increase in the endothermic peak from 92.56 °C to 120.39 °C, while the enthalpy increased from 495.86 Jg^−1^ to 504.21 Jg^−1^. The untreated 30 wt% PA/FL with 2% CMF presented lower enthalpy and endothermic peak due to the poor bonding capacity of untreated cellulose fibers.

Souza et al. [136] studied the effect of the percentage of fiber on the thermal characteristics of natural composites reinforced with caranan fiber. The studied variation of fiber/matrix percentage was from 0% to 30%. The authors show that the increase of the caranan fiber increases the *T_g_* of the composites. The fiber contents with 20% and 30% had a *T_g_* value of 96 °C and 113 °C, respectively. Devireddy et al. [137] studied the thermal properties of banana/jute epoxy hybrid composites. The transverse and longitudinal thermal conductivities of the hybrid composites decreased by 44.35% and 34.98%, respectively, for 30 wt% jute and 10 wt% of banana fibers specimens. The thermal diffusivity and specific heat capacity of jute/banana hybrid composite decreased with increasing the fiber content. The combination of jute (30 wt%) and banana (10 wt%) presented the highest thermal stability at a higher temperature range.

## 3. Methods Used to Determine the Thermal Properties of Natural and Hybrid Composites

**Figure 2:**
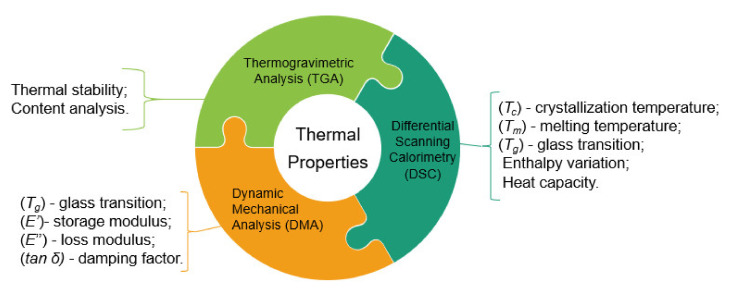
Schematic of the principal methods used to determine the thermal properties of composites. Reproduced with permission from [35].

Different methods are used to determine the thermal properties of natural and hybrid fiber composites and to help understand and determine the suitability of different fiber-reinforced composites for a certain application. The methods used in the literature for thermal analysis of composites are as follows: Thermogravimetric analysis (TGA), differential scanning calorimetry (DSC), and dynamic mechanical analysis (DMA). Figure 2 presents a summary of the main methods used to determine the thermal properties of composite materials with the main thermal properties of the composites provided by these techniques. This section presents an overview of the main techniques used for the thermal characterization of natural and hybrid fiber composites.

### 3.1. Thermogravimetric Analysis (TGA)

TGA analysis consists of measuring the weight of a material sample as a function of temperature and/or time, in a specific controlled atmosphere (e.g., nitrogen, helium, air, or other gases) [74,138,139]. Different temperatures and measurement times are applied in accordance with the matrix type of the natural fiber composite sample [39]. Typical thermogravimetric parameters consist of moisture, volatile substances, loss on ignition, or ash. The thermal data obtained from TGA analysis are dependent on several parameters, such as the sample’s mass and form, atmosphere, flow rate, heating rate, and the treatment applied [39]. For example, sample mass and form affect the profile of the TG curve. A big sample will possibly present thermal gradients in the sample, which will determine a temperature deviation from the set temperature due to endo- or exothermal reactions as well as a delay in mass loss.

As discussed in the previous section, the thermal behaviour of natural fiber composites depends on the principal constituents (natural fibers and matrix type) of the composite. For example, similar natural fibers, such as wood, jute, and sisal, present similar TG/DTG curves and thermal decomposition patterns. In general, the derivative thermogravimetric test (DTG) curve of natural fibers shows elimination of water and the thermal decomposition of cellulose components of the fibers. The DTG curve shows the maximum rate of peak thermal decomposition, and fiber constituents are indicated by peaks in each degradation range. For instance, the lignocellulose fibers (i.e., jute, flax, wood, sisal, ramie, curauá etc.) present three stages of degradation. The first stage is related to water loss by natural fiber (60–100 °C). The second stage is related to the loss of the main constituents of the fibers: hemicellulose, cellulose, and lignin (200–500 °C). Finally, the last stage of degradation is the formation of active coal as a form of residue [48,140,141,142,143].

Table 2 summarizes several studies which present the stages of decomposition of different natural fiber reinforced composites.

Several authors used the thermogravimetric analysis to determine the thermal properties of natural fiber composites [52,53,84,85,104,107,128,150,151,152]. For example, Boopalan et al. [153] studied the thermal properties of jute (J)/banana (B) reinforced epoxy composites. The TGA analysis showed that the combinations of J (50%)/B (50%), J (75%)/B (25%), and J (25%)/B (75%) presented initial weight loss at 200 °C due to solvent removal in the epoxy matrix and major weight loss occurs at 380 °C (75.64%), 377.72 °C (82.14%), and 376.51 °C (79.01%), respectively. However, the J/Epoxy and B/Epoxy presented the initial weight loss at 190 °C because of solvent removal from the matrix and major weight loss at 376.51 °C (79.01%) and 377.72 °C (74.43%), mainly due to epoxy and fiber volatilization and degradation. Therefore, the 50% jute and 50% banana fiber epoxy hybrid composite showed better thermal properties than other combinations.

**Figure 3:**
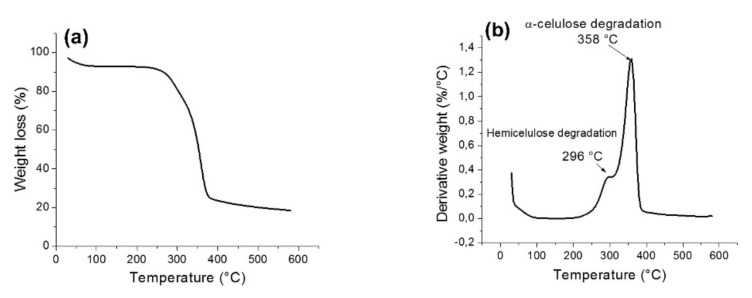
(**a**) TG and (**b**) DTG curves of Fique fibers at heating rates of 10 °C/min. Reproduced with permission from [145].

Hidalgo–Salazar et al. [145], studied the thermal properties of fique fiber reinforced natural composites. Two types of resins (linear low-density polyethylene (LLDP) and epoxy resin (EP)) were used. Figure 3 presents an example of representative TG (Figure 3a) and DTG (Figure 3b) curves of the Fique fiber, which show that degradation occurred at 296 °C, while the neat EP starts its decomposition at 96 °C. Therefore, the EP/Fique samples degraded after the neat resin in both degradation steps. This has been previously reported in the available literature regarding EP/Phormium tenax leaf fiber composites and was explained to be due to an enhanced fiber/matrix interface [154]. The residual char after final degradation was 6.1% for neat epoxy resin and 11.1% for epoxy-Fique natural fiber composite. The authors state that this increase in the residual char of the composite was due to the Fique fiber incorporation in the epoxy resin.

**Figure 4:**
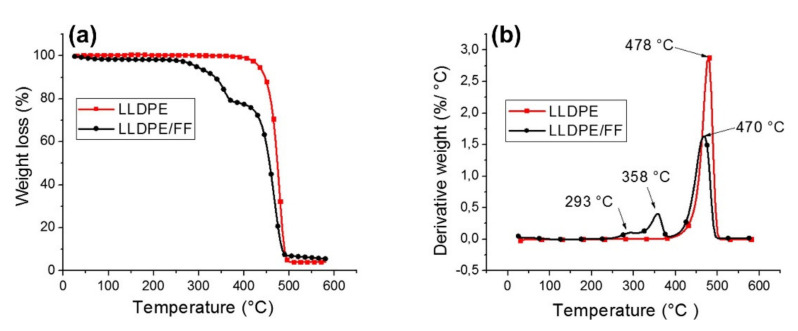
(**a**) TG curve of neat Linear Low-Density Polyethylene (LLDPE) and (**b**) DTG curve of Linear Low-Density Polyethylene nonwoven Fique Fiber natural composite (LLDPE/FF). Reproduced with permission from [145].

**Figure 5:**
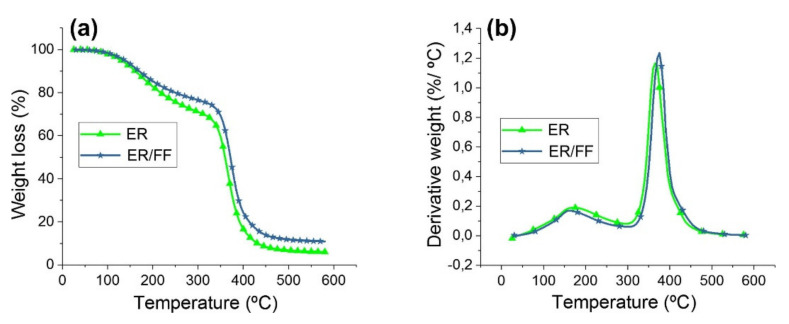
(**a**) TG and (**b**) DTG curves of neat Epoxy resin (EP) and Fique biocomposite based Epoxy (EP/FF). Reproduced with permission from [145].

Figure 4 presents an example of representative TG and DTG curves of LLDPE, LLDPE/Fique, while Figure 5 shows the thermal curves for EP and EP/Fique natural composites, respectively. For the neat LLDPE sample a single step degradation was observed with *T_o_* (onset) at 439 °C and *T_max_* and 478 °C, respectively (see Figure 4a). The char residue at the end of the degradation was 4.1%. For the PE/Fique case, a two-step degradation process was observed. The authors state that the first degradation step is linked to the fiber constituent degradation (*T_o_* at 266 °C) presenting a mass loss of 21%. A minor decrease on the thermal stability of the polymeric matrix was observed, which can be linked to the degradation of fibers on the LLDPE during the thermocompression process at 170 °C. Moreover, the residual weight of LLDPE-Fique increased to 5.6%, due to the Fique fiber addition. In Figure 4b, it can be seen that the DTG curve presented two *T_max_* peaks at 293 °C and 358 °C, which can be linked to hemicellulose and α-cellulose degradation. The second degradation step is related to LLDPE matrix decomposition. The process starts at 437 °C and presented a *T_max_* of 470 °C. On the other hand, a two-stage weight loss process was reported for both EP and EP/Fique composites, indicating similar thermal degradation behaviour (see Figure 5). The first degradation step (90 °C to 200 °C) is related to small molecular decomposition of EP. For this stage, the reported *T_o_* were 96 °C and 114 °C, while *T_max_* were 167 °C and 165 °C for EP and EP/Fique, respectively. In addition, the second degradation step (250–500 °C) illustrates the decomposition of the main polymeric chain. In this step, the observed *T_o_* was 344 °C and 352 °C while *T_max_* was 365 °C and 373 °C for EP and EP-Fique, respectively.

**Figure 6:**
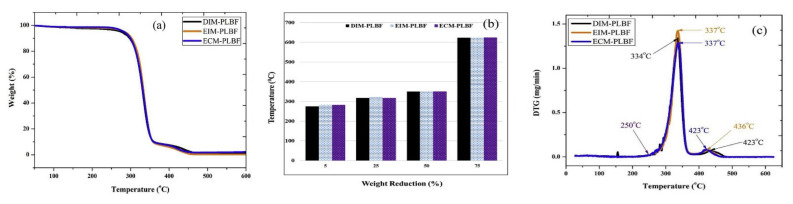
(**a**) TGA (**b**) mass reduction and (**c**) DTG curves of banana/PLA based composites fabricated using three processing techniques (DIM, EIM and ECM). Reproduced with permission from [156].

Neher et al. [155], investigated the thermal properties of the palm fiber reinforced composite of acrylonitrile butadiene styrene (ABS). The composites were manufactured using an injection moulding machine in different weight fractions: 5, 10, and 20 wt%. They found that thermal degradation occurs only in one stage for ABS (100%), while the ABS composite with 10% of fiber presented higher *T_onset_* when compared to other reinforced cases. In a recent study, Komal et al. [156] investigated the thermal properties of short banana fiber/PLA fabricated by different processing techniques (injection molding (DIM), extrusion injection molding (EIM), and extrusion compression molding (ECM)). In Figure 6a, it can be seen that all the composites exhibited a similar trend of thermal decomposition. Figure 6b shows that all the composites lost 5%, 25%, 50%, and 75% of their weight in the temperature range of 275–285 °C, 315–320 °C, 350–355 °C, and 620–625 °C, respectively. Similar to the other studies discussed above (see Table 1), the loss of weight of the composites studied can be divided into three stages: 1. The loss of weight at around 80–160 °C attributed to the evaporation of moisture present in the banana fiber; 2. The weight reduction in the temperature range of 250–350 °C due to the decomposition of hemicellulose, cellulose, and some portion of the lignin of the banana fiber and pyrolysis of the PLA; 3. The weight loss at the temperature range of 350–450 °C due to the pyrolysis of remaining lignin and pyrolysis of the residue of lignin and PLA. DTG curves (Figure 6c) show the precise peak position of the degradation temperature (maximum reduction in weight between 250 °C and 350 °C), which represents the pyrolysis of the hemicellulose and cellulose of the fiber and PLA.

Table 3 summarizes the results from several recent studies on the thermal properties of natural fiber composites.

### 3.2. Differential Scanning Calorimetry (DSC)

The DSC determines the material transitions as a function of temperature and time. The endothermic (heat absorption) and exothermic (heat released) peaks and magnitudes indicate the thermal phase transformation of the composites [162,163]. The principal thermal data extracted from this analysis are the glass-transition temperature (*T_g_*), degree of crystallization (*X_c_*), crystallization temperature (*T_c_*), and fusion temperature (*T_m_*). The enthalpy variation and heat capacity of the composite can also be determined.

The *T_g_* is an important material property when considering the natural composites for a particular end-use application. *T_g_* is the temperature band in which a thermoset polymer shifts from a stiff to a more flexible or rubbery state. It is well known that the “normal” state of most thermoset polymers at room temperature is rigid (amorphous solid). Below the *T_g_*, the molecular chains of the thermoset resins do not present enough energy to let them move around (the molecules are frozen in place as a rigid structure because of the short chain length, molecular groups separating off the chain, and interlocking with each other). Moreover, when the polymer resin is heated, the molecules of the polymer resin gain energy and they can start to move around. The amorphous rigid structure of the thermoset polymer resin is transformed to a flexible structure (rubbery state) when a certain heat energy level is attained, and the polymer molecules are allowed to move freely around each other. This transition point is termed the glass transition temperature. To conclude, the service temperature of polymer resins should be always below the *T_g_*. If the composites are used above their *T_g_*, they will quickly lose their mechanical properties (strength and stiffness), and they will continue to maintain some mechanical properties until the temperature reaches *T_m_*. The crystallization temperature (*T_c_*) is associated with the point where polymer chain alignment modification is possible. Upon reaching the *T_c_*, ordered crystalline chain regions appear, called lamellae. However, amorphous regions still remain in the structure. It should be noted that the crystallization is an exothermic peak in a DSC curve. *T_c_* temperature is higher than *T_g_* but still lower than that of the melting temperature, (*T_m_*). Finally, the melting temperature (*T_m_*) is the point where the polymeric chains lose their bonds and turn into a liquid. This process is called endothermic transition. In general, *T_m_* for a thermoset polymer is higher than its *T_g_*. At temperatures above *T_g_* but below *T_m_*, the polymer resin is in the rubbery state and the material can exhibit large deformations under a relatively low load.

There are several factors that can affect the data collected by the DSC analysis, such as sample size and shape, heat ramp, and type of atmosphere. Several authors used the DSC technique to investigate the thermal stability of natural fiber and hybrid composites [66,86,107,116,134,136,159,164,165,166]. For instance, Gupta et al. [164], used the DSC technique to investigate the effect of hybridization on the thermal properties of jute/sisal fibers in epoxy-based composites. The studied cases were: jute (J1), sisal (S1), 50% of jute + 50% of sisal (H1), 25% of jute + 75% of sisal (H2) and 75% of jute + 25% of sisal (H3). The composites were manufactured using the hand-lay-up technique and total fiber loading of 30%wt. They found that hybridization positively affected the *T_g_* of the composites. The values of *T_g_* found for H1 samples was 73.86 °C, for the H2 case was 72.86 °C, while for H3 the *T_g_* value found was 68.36 °C when compared with the *T_g_* of the matrix (65.16 °C). Moreover, the exothermic temperature (*T_d_*) for hybrid composites and natural fibers presented higher values than the pure matrix.

**Figure 7:**
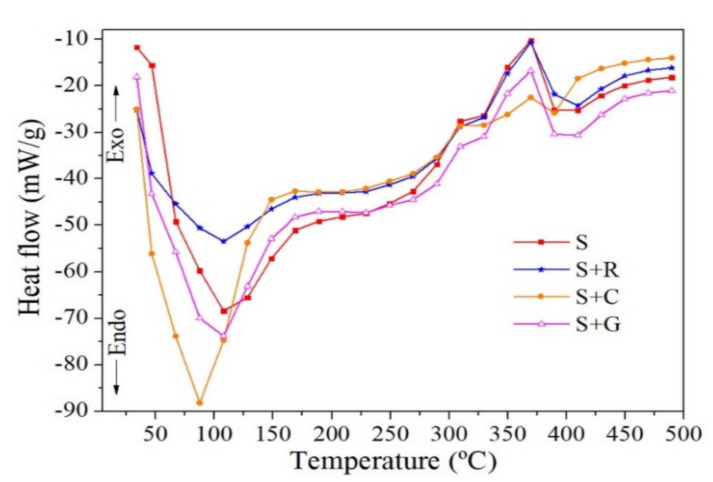
DSC curves of epoxy sisal based composites as a function of hybridization (S-Sisal; S + R-Sisal + ramie; S + C-Sisal + curauá and S + G-sisal + glass fiber), Reproduced with permission from Pereira et al. [107].

Pereira et al. [107] used DSC to investigate the influence of the hybridization on the thermal properties of pure sisal and epoxy hybrid composites. Figure 7 shows the DSC curves for the composites studied. It can be seen that two events predominate, endothermic and exothermic around 100 °C and 375 °C, respectively. Sisal + curauá was the sample that absorbed the greatest heat in the endothermic event and the one that least released heat in the exothermic event.

Table 4 summarizes the results from several recent studies on the thermal properties of natural fiber composites obtained from DSC analysis.

### 3.3. Dynamic Mechanical Analysis (DMA)

DMA determines the following thermal data: storage modulus (*E′*), loss modulus (*E″*), damping factor (*tan δ* = *E″/E′*) and glass transition temperature (*T_g_*) [173]. The storage modulus (*E′*) is associated with the energy storage of the elastic characteristics of the material [66,111,174,175]. It decreases with increasing the temperature and is associated with “stiffness” of the composites sample [111,174]. The loss modulus (*E”*) is linked to the energy dissipation promoted by the viscous part of the composite sample. This dissipation is related to the internal molecular friction of the molecular chains due to the following factors: morphological transformation and relaxation, morphology, and system heterogeneity [39,111]. The damping factor is defined by dividing the storage and loss modulus (*tan δ= E″/E′*) and is associated with the internal mobility of the polymeric molecular chains, showing the influence of the fiber/matrix interactions [65,111,175,176]. A high *tan δ* value indicates that the system is dissipating more energy than it is storing due to the fiber–matrix interaction quality, while a low *tan δ* value suggests that the polymeric chain has lower mobility showing a good fiber/matrix interfacial interaction. Determining *T_g_* using DMA measurements of the complex modulus is usually performed as temperature increases with a constant heating rate.

In the DMA analysis, several different test method configurations are available (i.e., dual cantilever, single cantilever, three-point bending, torsion, shear, tension, and compression). However, the most common test method for composite materials is the three-point bending mode, as it removes the combined loading present in single or double cantilever modes and yields measurable strains in relatively rigid materials. Depending on the methodology used, significant variation of the glass transition temperature for a given material may be reported via the DMA analysis (up to 25 °C). The calculation method may also be more or less conservative, taking the *T_g_* via first inflection point/modulus drop onset or the *tan δ* peak, respectively [177].

The thermal properties of the natural fiber composites collected via DMA depends on the physical or structural arrangement of phases (interface), morphology and the nature of natural composite constituents. It was shown in the literature that the presence of fillers, fiber content and orientation, and the chemical treatment of the fibers affect the dynamic mechanical properties of a composite material [110,111]. The mode of testing also has an influence on the DMA tests results.

The DMA analysis was used by several researchers to determine the thermal properties of natural fiber composites [52,65,71,82,164,166,178]. For example, Chee et al. [179] used the thermomechanical analysis (TMA) and DMA analysis to investigate the effect of the hybridization of bamboo (B) and Kenaf (K) fibers on the thermal properties of various configurations in epoxy resin-based hybrid composites. The authors reported that the composite with 100% Bamboo obtained a value of storage modulus (*E’*) of 979 MPa, while the epoxy resin and 100% Kenaf had a value of 449 and 775 MPa, respectively.

**Figure 8:**
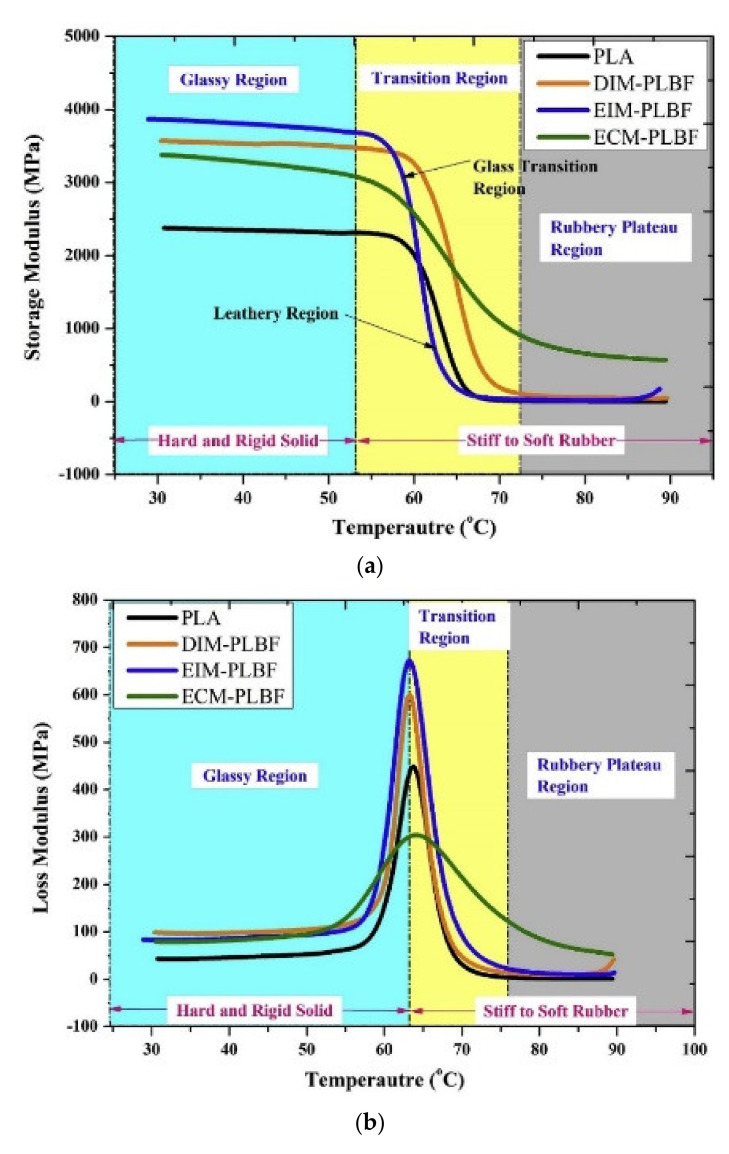

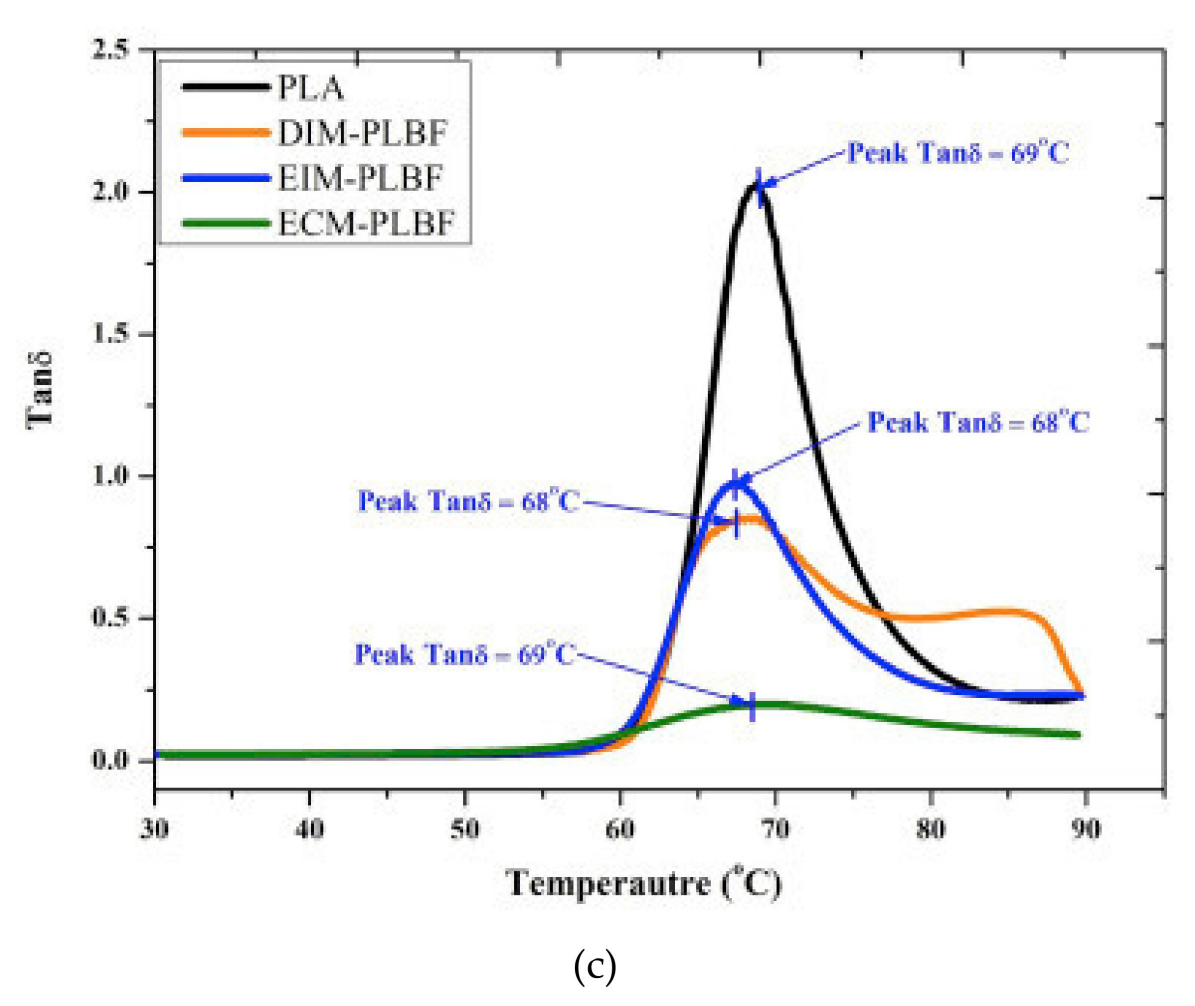
DMA test results of banana fiber/PLA composites (**a**) Storage modulus (E′) and (**b**) Loss modulus (E″), (**c**) Tan delta vs. temperature. Reproduced with permission from [156].

Komal et al. [156] used DMA to study the thermal properties of short banana fiber (20 wt%) and PLA matrix fabricated using different processing techniques (direct injection molding (DIM), extrusion injection molding (EIM), and extrusion compression molding (ECM)). They found that the dynamic mechanical properties (storage modulus, loss modulus, and tan delta) and crystallinity of the composites fabricated by EIM presented a significant improvement compared to the other fabrication techniques, as can be seen in Figure 8.

Table 5 summarizes the results from several recent studies on the thermal properties of natural fiber and hybrid composites obtained through DMA analysis.

## 4. Conclusions

Thermal analysis can provide useful information for the development of new materials and optimization of the selection process of these materials for new applications. The most common thermal properties studied in the literature are: the percentage of weight loss, the degradation temperature, *T_g_*, and viscoelastic properties (storage modulus, loss modulus, and the damping factor). Different factors affect the thermal properties of natural fiber composites (i.e., fiber and matrix type, the presence of fillers, fiber content, and fiber orientation, the chemical treatment of the fibers, manufacturing process, and type of loading). It is crucial to ensure that the natural fibers used in the composites can withstand the heat required during the fabrication process and retain their characteristics after exposure to heat. Different approaches were used in the literature for the enhancement of thermal properties of natural fiber-based composite materials. For example, using natural fibers with low lignin content leads to a better thermal performance of composites. Another approach involves the removal of lignin through fiber treatment. Finally, the incorporation of synthetic fillers or synthetic fibers in natural fiber reinforced composites increase their thermal stability.

## Figures and Tables

**Table 1:** Effect of treatments on the thermal properties of natural fiber-reinforced composites.

Fiber	Matrix	Treatment	Thermal Properties	Ref.
Flax	Epoxy	5% and 10% of sodium bicarbonate	By increasing the concentration of sodium bicarbonate, negligible changes in the *T*_g_ and reductions in the *tan δ* peak heights were found.	[81]
Mulberry (MF)	Polyester	Alkalization (5% ATMFC, 10% ATMFC and 15% ATMFC)	10% ATMFC samples presented higher values of storage modulus and loss modulus compared to the other cases studied.	[82]
Jute	Polyester	5% of alkali treatment, poly (lactic acid)-coated and mixed treatment	The mixed and alkaline treatment improved the *T_g_*.	[83]
Buriti and ramie	Polyester	NaOH (2.5 and 10%)	The buriti fiber starts to degrade at 217 °C, while the ramie fiber at 247 °C. The alkalization treatment of these fibers negatively affected their properties when compared to the in natura and washed cases.	[84]
Bamboo	Epoxy, polyester, and Vinyl ester	Chemical (10% NaOH) and physical (milling method)	The alkaline treatment enhanced the thermal stability of the composites.	[85]
Curauá	Polyester	10% of barium hydroxide Ba(OH)_2_ for 48 h at 25 °C, 14% of calcium hydroxide Ca(OH)_2_ for 4 h at 70 °C, 10% of potassium hydroxide (KOH) for 1 h at 25 °C, 5% of sodium hydroxide (NaOH) for 2 h at 70 °C and 5% of silane (Trimethoxy(propyl) for 4 h at 25 °C	The chemical treatment increased the *T*_g_ of the treated composite. The Ca (OH)_2_ treatment provided a *T*_g_ of 141.2 °C, compared to the 133.69 °C of the untreated composite.	[86]
Kenaf	PLA	Acetylation (0.5, 1, 2 and 3 h)	The thermal stability was improved as treatment time was increased.	[87]
Banana	PP	Untreated, alkaline and acetylated treatment	The acetylation treatment improved the thermal stability and raised up the crystallization temperature.	[88]
Jute	PLA	6% benzoyl peroxide acetone solution for about 30 min after alkali pre-treatment	Higher storage modulus and lower *tan δ* compared to untreated composite.	[89]
Kenaf	PU	Acetylation, blocked isocyanate, maleic anhydride and permanganate treatment	The acetylated treatment provided better values for storage modulus (E’) when compared to all other treatments.	[90]
Banana + sisal	PLA	Untreated and benzoyl peroxide treatment	The peroxide treatment improved the thermal stability by delaying the thermal degradation. No significant changes in the *T_g_* and melting temperature (*T_m_*).	[91]
Jute + curauá	Epoxy	Untreated, alkali, silane and mixed treatment	The chemical treatment improved both *T_g_* and thermal stability of the composites. *tan δ* value decreased.	[65]
Bamboo	Epoxy, polyester and vinyl ester	Alkali (0 h-0%, 24 h-10 %, 48 h-5, 10, 15% and 72 h-10%)	The bamboo-epoxy composite treated with the 10% of NaOH for 48 h presented the highest thermal stability.	[85]
Cotton	LDPE	Alkaline, silane, mixed treatment, maleic anhydride, and alkali–maleic anhydride	The mixed treated composite presented higher *T*_g_.	[80]
Jute	Epoxy	Enzyme treatment, ozone treatment and laser treatment	The laser treated composites presented higher *T*_g_ values in relation to the other treatments.	[92]
Pine cone	ABS	Alkaline and bleaching (alkaline + H_2_O_2_)	The NaOH treatment increased the *T*_g_ of ABS/FT2% and ABS/FT5% composite when compared to the pure ABS composite. The untreated and ABS/FB2% composites showed no significant change in *T*_g_.	[93]
Ramie	PLA	Maleic anhydride (MA)	The thermal stability increased but the *T_g_* decreased	[94]

**Table 2:** Stages of thermal degradation of natural fiber reinforced composites.

Fiber Type	Matrix	Stage 1	Stage 2	Stage 3	Ref.
Sisal, Sisal + Ramie, Sisal + Curauá	Epoxy	30–150 °C: evaporation of humidity retained in the fibers	240–420 °C: pyrolysis process.	-	[107]
Bamboo	Epoxy, polyester, vinyl ester	30–155 °C: evaporation of moisture.	199–399 °C: the decomposition of cellulosic components (cellulose and hemicellulose).	364–499 °C: the decomposition of lignin.	[85]
Sugar Palm	Phenolic	30–200 °C: corresponds to vaporization of water molecules	200–300 °C: the thermal degradation of the hemicellulose, cellulose and lignin.	300–400 °C: the loss of small groups and water bonds in the chains of the chemical structures.	[53]
Ramie and Buriti	Polyester	61 °C (Ramie)–69 °C (Buriti): is attributed to the water evaporating.	289 °C (Ramie)–292 °C (Buriti): The decomposition of amorphous constituents, such as hemicellulose.	368 °C (Ramie)–341 °C (Buriti): Thermal decomposition of cellulose.	[84]
*Arundo donax* L.	Benzoxazine resin	-	200–300 °C: The decomposition of hemicellulose and cellulose.	350–500 °C: The decomposition of lignin.	[144]
Fique	Linear Low-Density Polyethylene (LLDPE) and Epoxy	60–100 °C: The evaporation of superficial water.	250–350 °C: The decomposition of hemicellulose.	350–600 °C: The decomposition of α-cellulose.	[145]
Coir fiber	PLA	25–150 °C: attributed to the evaporation of water.	190 °C and 290 °C: corresponds to the hemicellulose degradation.	290 °C and 360 °C: the thermal degradation of cellulose. The degradation of lignin occurred between 280 °C and 500 °C	[146]
Kenaf and Rice husk	PLA	30–150 °C: The evaporation of the moisture absorbed in fibers.	230–350 °C: The degradation of the cellulosic substances of hemicelluloses, cellulose and lignin.	-	[147]
Sisal filler	Polyurethane (PU)	-	297 °C: the hemicellulose degradation.	365 °C: corresponded to cellulose degradation	[148]
*Carpinus betulus* L.	Polypropylene (PP)	-	223–290 °C: the hemicellulose degradation.	290–380 °C: the cellulose and lignin degradation	[149]

**Table 3:** Thermal properties of natural fiber composites obtained from TGA analysis.

Fiber	Matrix	Thermal Properties	Ref.
Thermoset matrices			
Bamboo	Epoxy, Polyester and Vinyl ester	The incorporation of bamboo fiber did not present a substantial improvement in the initial onset degradation temperature (*T*_onset_) of the composites.	[85]
Fique	Linear Low-Density Polyethylene (LLDP) and Epoxy resin (EP)	The incorporation of the fique fiber in LLDP matrix decreased the *T_onset_* and *T_max_* when compared to pure LLDP composite. For the EP, the fiber incorporation increased in *T_onset_* and *T_max_* compared to the pure epoxy case.	[145]
Buriti and ramie	Polyester	The maximum peak of degradation temperature (*T_d_*) for the ramie fiber reinforced composite was 372 °C, while for the buriti composite it was 346 °C.	[84]
Sisal, sisal + curauá and sisal + ramie	Epoxy	The hybridization increased the thermal stability of the composites when compared to the pure sisal composites.	[107]
Banana, jute Banana + jute	Epoxy	The thermal diffusivity and specific heat capacity of jute/banana hybrid composite decreased with increasing the fiber content.	[137]
Sugar Palm	Phenolic	The chemical treatment negatively affected the thermal stability of the composite.	[53]
Sisal and kenaf	Polyester	The thermal stability of hybrid composites was superior when compared to the neat fiber case	[150]
Sugar palm fiber (SPF) and roselle fiber (RF)	Polyurethane	The sugar palm fiber (SP) improved thermal stability of hybrid composite. The roselle (25 wt%) + sugar palm (75 wt%) composite showed higher thermal stability when compared to RFT composite.	[104]
Jute, jute + sisal and jute + curauá	Epoxy and Polyester	*T*_onset_ was higher for the jute, jute + curauá epoxy composites compared to polyester composites. For jute + sisal there was no significant change in *T*_onset_ for both matrices.	[19]
Kenaf + pineapple	Phenolic	The treatment increased the maximum degradation temperature (*T_d_*).	[52]
Curauá	Polyester	The addition of the fiber and chemical treatment of fibers with NaOH improved the thermal stability of the composites.	[86]
Mulberry	Polyester	The thermal stability of the composites increased by increasing the NaOH concentration.	[82]
Jute + Oil palm	Epoxy	The hybridization of the composites increased the maximum degradation temperature when compared to the pure Oil palm composite.	[141]
Date palm fibers (DPF)	Epoxy	DPF improves the thermal stability of the composite. *E*′ and *E*″ increased (50% DPF presented higher improvement compared to 40% and 60% DPF content).	[157]
Thermoplastic matrices			
Banana	ABS, high impact polystyrene (HIPS) and HDPE	The addition of natural fiber to the thermoplastic matrix showed an increased thermal stability when compared to the pure resin.	[110]
Date palm	PVC and HDPE	The chemical treatment improved the *T_onset_* and *T_max_* of the date palm reinforced composites when compared to the untreated composite and the pure matrix.	[70]
Basalt + Cissus quadrangularis	PLA	The addition of basalt fiber increased the thermal stability of the composite.	[158]
Jute	PLA	The configuration 2J5P-2 (2 layers of jute and 5 layers of PLA) of composite showed an increase in *T_onset_* and *T_max_* when compared to the other cases.	[152]
Wood powder	Polypropylene (PP)	The incorporation of wood powder presented maximum temperature (482.3 °C) for 45 wt% when compared to pure PP composite (475.3 °C).	[159]
Flax + Basalt	PLA	The hybridization of the composite showed an increase in *T_onset_* when compared to the flax + PLA composite. The increment of basalt fiber increased the thermal stability of the composite.	[151]
Banana	PLA	The presence of banana fibers in PLA matrix led to a reduction in degradation temperature as compared to neat PLA, which was attributed to the low thermal stability of banana fibers that possibly enhanced deformation of the crystalline structure of PLA at higher temperatures.	[57]
Jute + Maleic anhydride (MAPP)	Polypropylene (PP)	The incorporation of MAPP did not have significant influence on the thermal stability of the composites.	[160]
Kenaf + epoxidized jatropha oil (EJO)	PLA	The incorporation of EJO (5 wt%) slightly increased the thermal stability (*T_onset_* and *T_end_*) of composites when compared to EJO (1 wt%).	[161]
Sisal	Polyurethane (PU)	The chemical treatment improved the thermal stability when compared with untreated composite.	[148]
*Carpinus betulus* L.	Polypropylene (PP)	The addition of fiber lowered the thermal stability when compared with pure PP matrix.	[149]

**Table 4:** Thermal properties of natural and hybrid composites obtained from DSC analysis.

Fiber	Matrix	Thermal Properties	Ref.
Thermoset Matrices			
Curauá	Polyester	The chemical treatments used increased the *T_g_* of the composites. The best treatment was Ca (OH)_2_ with a *T_g_* value of 141.92 °C.	[86]
Jute + ZrO_2_	Polyester	The presence of the nanofiller increased the *T_g_* of the composite.	[165]
Jute+ ramie	Epoxy	Alkalization and mixed (alkalization + silane) treatment increased the thermal properties.	[65]
Caranan	Epoxy	The endothermic peak shows a large amount of water retained in the fiber.	[136]
Jute + sisal	Epoxy	The addition of natural fiber produced an increase of thermal properties (*T_g_* and *T_c_*).	[164]
Hemp+ eggshell	Epoxy	The incorporation of filler reduced the exothermic peak of the composite.	[134]
Flax + Pineapple + Micro Cellulose (CMF)	Epoxy	The addition of CMF improved the endothermic peak and enthalpy when compared to the unmodified composite.	[116]
Kenaf + Sisal	Bio-Epoxy	UV aging increased the *T_g_* of hybrid composites and pure fibers.	[166]
Jute + coir	Epoxy	The endothermic peak showed water loss between 60–120 °C.	[167]
Flax + TiO_2_	Epoxy	The addition of 0.7% of nanofiller increased the *T_g_* by 5 °C when compared to the unfilled composite.	[168]
Thermoplastic matrices			
Cotton	Low-density polyethylene (LDPE)	The mixed (alkali–silane) treated composite presented higher values for *T_g_* when compared with other chemical treatments.	[80]
Bamboo	Polypropylene (PP)	The incorporation of natural fiber and nanofiller (TiO_2_) improved the crystallization temperature (*T_c_*) when compared to pure PP composite.	[169]
Pine cone (*Pinus elliottii*)	ABS	The chemical treatment (NaOH) increased the *T_g_* of the composite when compared to pure ABS composite.	[93]
Wood powder	Polypropylene (PP)	The addition of wood powder (15 wt% and 30 wt%) showed no significant change for the melting temperature (*T_m_*). However, the composite with 45 wt% presented a decrease of 2 °C in *T_m_*.	[159]
Bamboo	PLA	The alkalinization treatment of bamboo fiber increases thermal stability (*T_g_* and *T_c_*) when compared to untreated composites.	[170]
Date palm	PVC and HDPE	The chemical treatment (H_2_O_2_+ HNO_3_) increased the *T_g_* of the date palm reinforced composite when compared to the other treatments and the pure matrix.	[70]
Kenaf fiber; up to 10% (*w/w*) of thymol	PLA	No change in *T_g_* with an increase in kenaf fiber content unless when plasticizer is used. A decrease in all of the key thermal transitions with the addition of 5% and 10% (*w/w*) thymol into the neat PLA and PLA/kenaf composites was found.	[171]
Coir fibers	PLA	Addition of coir fibers increased the *T*_g_ and degree of crystallinity while the crystallization temperature decreased.	[172]
Coir fibers	PLA	Addition of coir fibers in PLA matrix does not affect the *T_g_* and melting temperatures of the coir fiber-reinforced PLA. However, the cold crystallization temperatures decreased with increasing fiber content	[146]
Basalt + *Cissus quadrangularis*	PLA	The PBC2 (PLA + 12 wt% of basalt + 0.5 wt% of *Cissus quadrangularis*) composite showed maximum *T_g_* = 58.72 °C and *X_c_* = 24.43%.	[158]

**Table 5:** Thermal properties of natural composites obtained from DMA analysis.

Fiber	Matrix	Thermal Properties	Ref.
Thermoset Matrices			
Jute + sisal	Epoxy	The hybridization increased the storage modulus (*E*′) of composites. *T*_g_ of the hybrid composites was lower compared to pure jute composite.	[164]
Kenaf	Polyurethane (PU)	The acetylated treatment presented better values for storage modulus (*E*′) when compared with all other treatments.	[90]
Pineapple + kenaf	Phenolic	The treated pineapple fiber increased the *T*_g_ of the composites.	[52]
Mulberry	Polyester	10% of NaOH treatment increased the storage modulus (*E*′) of the composite. The fibers decreased the *T*_g_ (values of 69 °C was found for the neat resin and 63 °C was found for the untreated composites).	[82]
Aloevera /Hemp/Flax	Epoxy	The hybridization and chemical treatment (BaSO_4_) increased the storage modulus (*E*′) and *T_g_* of composites.	[180]
Kenaf + Nanofiller CNFs	Epoxy	The incorporation of nanofiller improved the *T_g_* of the composites and improved thermal stability and residual char of kenaf/epoxy composites.	[181]
Kenaf (KKK), Sisal (SSS) and Kenaf + Sisal (KSK and SKS)	Bio-Epoxy	The incorporation of fibers (sisal and kenaf) did not affect the *T_g_* of the composites. The hybridization increased the values of storage modulus (*E*′) when compared to pure resin.	[166]
Jute + Nanoclay	Epoxy	The nanoclay modified jute composites presented higher *E*′, *E*″ and *tan δ* values. The composites modified with 5 wt% of nanoclay had improved viscoelastic properties. 5% treated jute fiber composites presented the highest *E*′ and *E*″ values but the lowest *tan δ* value. 5% NaOH + 5 wt% nanoclay specimens presented higher storage modulus and *T_g_*.	[71]
Bamboo + Kenaf + Nanoclay	Epoxy	The addition of the nanofiller improved the storage modulus, loss modulus and *tan δ* when compared to the other hybrid composite.	[182]
Ramie + Buriti	Polyester	The ramie reinforced composite treated with 2% de NaOH presented higher storage modulus and loss modulus compared to the other treated cases.	[84]
Jute + Oil palm	Epoxy	High oil palm to jute fiber ratio lowered the storage modulus. Loss modulus presented an increasing trend as a function of increasing jute fiber content.	[141]
Bamboo + Kenaf	Epoxy	The complex and storage modulus of bamboo composite are higher compared to kenaf composite. Hybrid composites value lie between bamboo and kenaf composites.	[179]
Date palm + Bamboo	Epoxy	The hybridization of the composite presents an increase of storage modulus (*E*′) when compared with single fiber composites.	[69]
Buriti	Epoxy	The addition of buriti fiber improved the storage modulus (*E*′) and *T_g_* when compared with pure epoxy.	[183]
Thermoplastic matrices			
Flax + Basalt	PLA	The addition of natural fiber increased the storage modulus (*E*′) of the composites when compared to pure PLA composite.	[151]
Date palm	PVC and HDPE	The chemical treatments increased the properties of *T_g_* and storage modulus (*E*′) when compared to untreated composites and pure matrix.	[70]
Sisal	PLA	The sisal fiber with 15% weight fraction increased the thermal properties with maximum storage (*E*′ = 3430 MPa) and maximum loss moduli (*E*″ = 245 MPa).	[184]
Bamboo, wood and coconut	PLA	The addition of natural fibers increased the crystalline degree of PLA matrix. Also, increased the storage modulus (*E*′) when compared with pure PLA.	[185]
sugar palm fiber (SPF)- and kenaf fiber (KF)-	Polypropylene (PP)	The hybrid composite with the best ratio (PP/SPF/KF), T-SP5K5, showed a loss modulus (*E*″) of 86.2 MPa and a damping factor of 0.058.	[61]
Ramie	PLA	The chemical treatments (NaOH and Silane) showed maximum storage modulus (*E*′) when compared to untreated composite.	[186]
Coir + pineapple leaf	PLA	The hybridization of the composite showed an increase of storage modulus (*E*′) and loss modulus (*E*″) when compared to pure PLA.	[187]
*Carpinus betulus* L.	PP	The incorporation of nanofiller increased the storage modulus (*E*′) and *T_g_* when compared with pure PP.	[149]
Kenaf	PLA	The randomly oriented (ROFRPC) composite showed highest storage modulus (*E*′) when compared with unidirectional (UDFRPC) and bidirectional (BDFRPC) kenaf composites. The incorporation of different kenaf mats with PLA improved the *T_g_* of the composites as compared to neat PLA. ROFRPC showed the highest value of *T_g_*.	[188]
Basalt (BF)	PLA	The addition of BF improved the storage modulus (*E*′) when compared with pure PLA. Increasing the fiber content decreased *tan δ* (the presence of BF prevents the chain mobility of the pure PLA).The addition of BF had little influence on the *T_g_* and *T_m_* of the PLA composites.	[189]
